# Case Report: Resolution of negative dysphotopsia after capsulorhexis revision in an extended depth of focus intraocular lens

**DOI:** 10.3389/fopht.2026.1852810

**Published:** 2026-07-01

**Authors:** Rafael Lucena, David Goldblum, Tamer Tandogan

**Affiliations:** 1 Department of Ophthalmology, Pallas Klinik, Olten, Switzerland; 2 Medical Faculty, University of Basel, Basel, Switzerland; 3 Department of Ophthalmology, University of Heidelberg, Heidelberg, Germany

**Keywords:** capsulorhexis, cataract surgery, dysphotopsia management, EDOF IOL, intraocular lens, IOL intolerance, negative dysphotopsia

## Abstract

Negative dysphotopsia (ND) is a known cause of patient dissatisfaction following cataract surgery with intraocular lens (IOL) implantation. While IOL explantation is often considered in persistent cases, identifying reversible anatomical factors is essential to minimize surgical risk. Capsulorhexis size and configuration have been implicated in the pathogenesis of ND but remain underrecognized as a direct therapeutic target. A 73-year-old woman underwent uncomplicated phacoemulsification with implantation of an extended depth of focus (EDOF) IOL in the left eye. Postoperatively, she experienced persistent negative dysphotopsia despite optimal visual acuity, which significantly impacted her daily activities. At 6 months, she was referred to a university hospital where IOL explantation was recommended. On presentation to our department for a second opinion, slit-lamp examination revealed optimal IOL centration but a markedly small and irregular capsulorhexis. No posterior capsule defect, opacification, or refractive error was noted. The patient underwent surgical enlargement of the anterior capsulorhexis and removal of fibrotic capsular tissue using microsurgical scissors. The IOL remained stable, and no intraoperative complications occurred. Postoperatively, the patient reported progressive improvement in visual quality. At the 1-month follow-up, negative dysphotopsia had resolved, visual acuity remained stable, and patient satisfaction was high, avoiding the need for IOL explantation. Small or irregular capsulorhexis can be an underrecognized and reversible cause of IOL intolerance. Surgical revision of capsulorhexis may represent a safe and effective, lens-preserving alternative to IOL explantation in selected patients. Careful anterior segment evaluation should be considered in the diagnostic workup of postoperative dysphotopsia to identify candidates for this targeted intervention. This approach may be considered prior to IOL explantation in the management of persistent negative dysphotopsia.

## Background

Cataract surgery has become a routine procedure, leading to highly predictable postoperative outcomes and patient satisfaction (1). Recently, new lenses have come to the market, multifocal intraocular lenses (IOLs) and EDOF IOLs, being used to improve spectacle independence following cataract surgery (1, 2). However, a subset of patients experienced postoperative negative dysphotopsia (ND), as a dark shadow, and positive dysphotopsia (halos and glare). Usually, ND is commonly associated with a well-centered in-bag IOL. It may occur in both monofocal and multifocal designs, and the exact pathophysiology remains incompletely understood (3, 4). Recent evidence points to anterior capsulorhexis size and centration as major determinants of ND, where the anterior capsulorhexis edge overlaps the optic from the IOL, creating an illumination gap resulting in a shadowing perceived by the patient (5, 6). Also, an excessively large capsulorhexis may have a negative impact, as inadequate overlap of the intraocular lens optic leaves the lens edge exposed, which may contribute to the development of negative dysphotopsia. Other factors have also been proposed, such as the axial distance of the IOL behind the iris, a sharp or truncated edge, high index of refraction optic material (acrylic), haptic orientation, and a large κ angle (5, 6). In such cases, IOL explantation is often considered, carrying a burden not only for the surgeon but for the patient.

The role of capsulorhexis as a contributor to ND has not been well characterized, and reports describing isolated capsulorhexis revision as a lens-preserving alternative to IOL explantation in EDOF-related ND remain limited. We report a case in which intolerance to an EDOF IOL was successfully resolved by capsulorhexis correction, thereby avoiding IOL explantation.

## Case presentation

A 73-year-old female patient underwent uncomplicated phacoemulsification with implantation of an acrylic, squared-edge EDOF IOL (Vivinex Impress XC1, HOYA Surgical Optics, Japan) in the left eye at an external institution. Postoperatively, she complained of persistent ND in her left eye despite a distance visual acuity of 0.0 logMAR, which was disturbing her daily life.

At 6 months postoperatively, the patient continued to report persistent negative dysphotopsia (ND), which was interfering with her daily activities. The patient described a persistent dark crescent-shaped shadow in the temporal peripheral visual field of the operated eye, most noticeable under photopic and mesopic conditions. Symptoms were particularly pronounced in well-lit environments such as walking outdoors, computer activities, or transitioning between indoor and outdoor lighting. The visual disturbance significantly affected the patient’s quality of life. She reported constant awareness of the shadow during reading, computer use, and ambulation. Despite excellent measured visual acuity, the persistent subjective visual disturbance was considered functionally disabling. Given the impact on her quality of life, she was referred to a university hospital for further evaluation. After assessment, explantation of the EDOF intraocular lens (IOL) was recommended in response to her ongoing symptoms. Seeking a second opinion, she presented to our institution.

**Figure 1:**
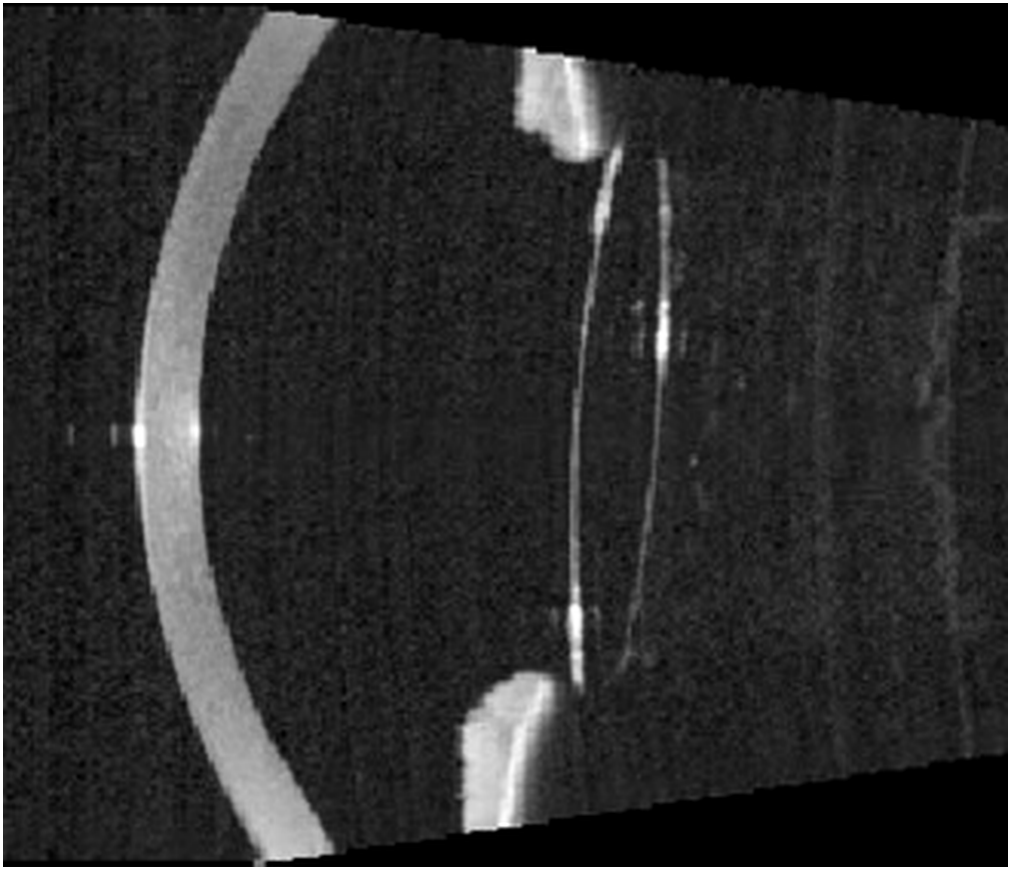
Centered IOL with optimal iris IOL distance.

**Figure 2:**
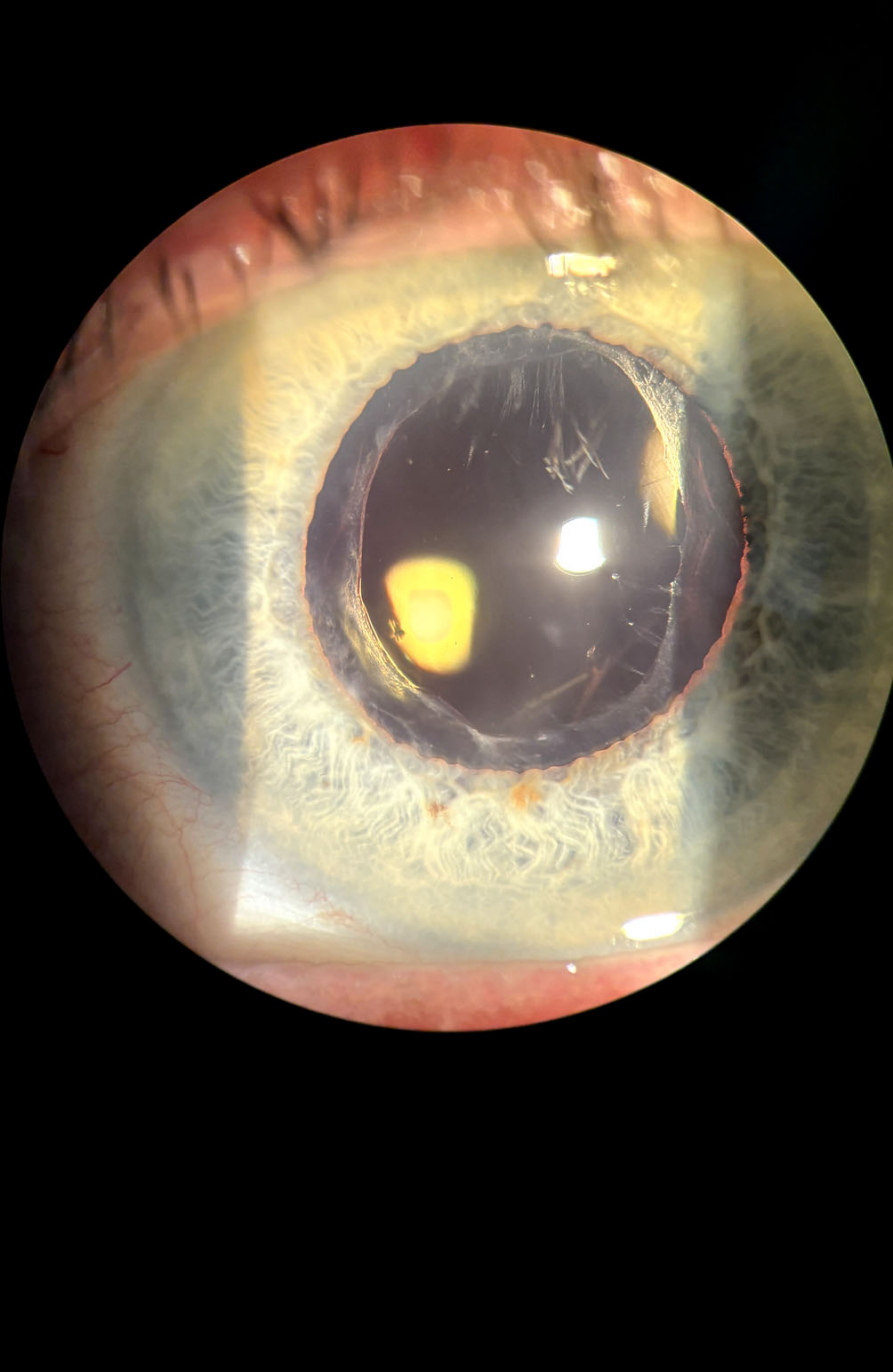
Preoperative capsulorhexis: small and irregular, overlapping the optic of the IOL.

Slit-lamp examination revealed an optimal distance iris to IOL (**Figure 1**) and an optimal centration of the IOL within the capsular bag. However, the capsulorhexis was markedly small and irregular, approximately between 4.5 and 5.0 mm (**Figure 2**). No posterior capsule defect, opacification, or clinically relevant refractive error was observed.

Given the anatomical findings, fibrotic ring removal and capsulorhexis enlargement were proposed as an alternative to IOL explantation.

Under standard sterile conditions, surgical enlargement of the anterior capsulorhexis and removal of fibrotic capsular tissue were performed with microsurgical Vannas scissors, resulting in a symmetric capsular opening. Intraoperatively, no single sector of the capsulorhexis was identified as exclusively responsible for the symptoms. The fibrotic areas were carefully removed, and the capsulorhexis was regularized and widened to approximately 5.5 mm during surgery. The IOL remained stable within the capsular bag throughout the procedure. No intraoperative complications occurred.

**Figure 3:**
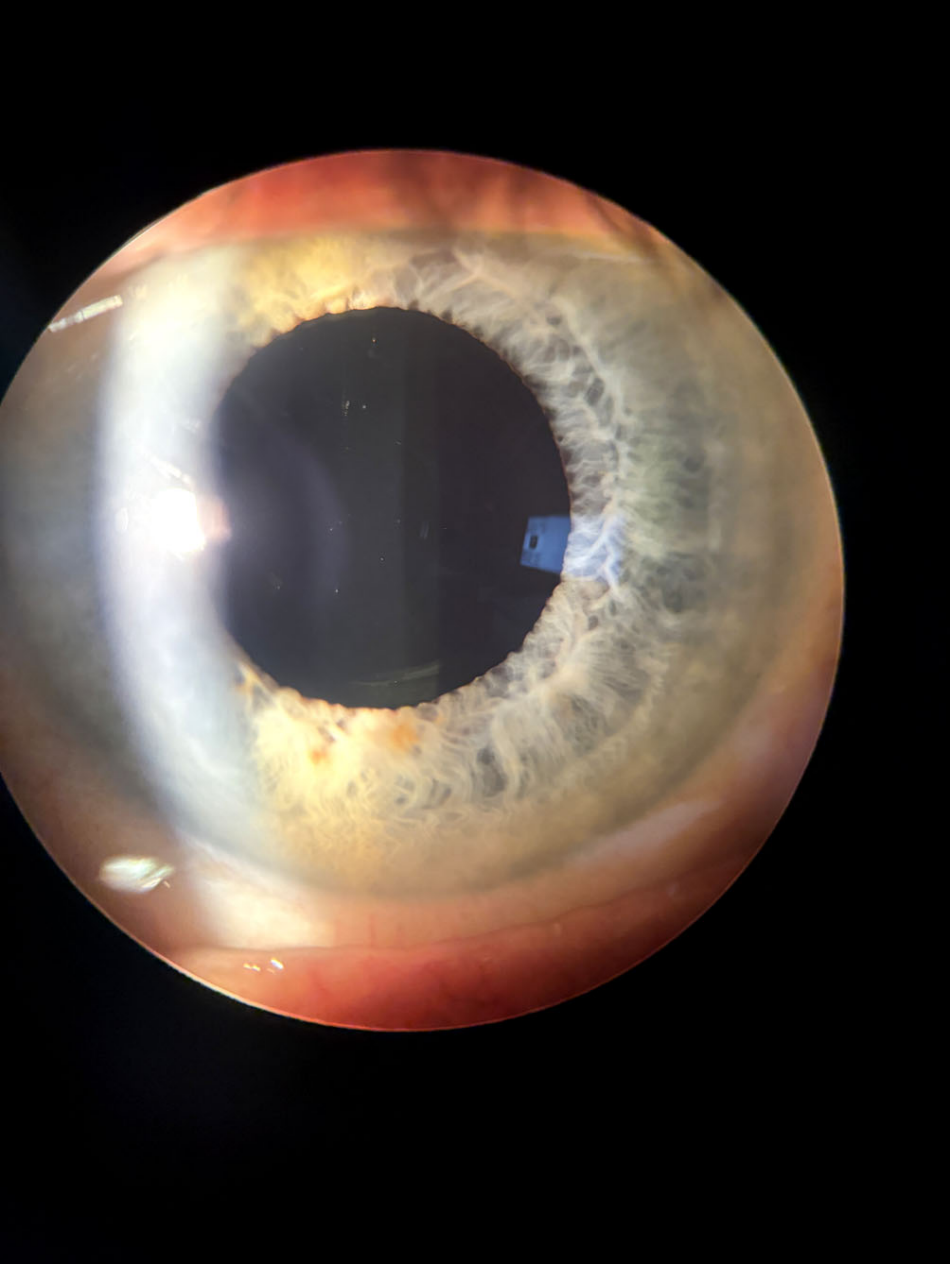
Capsulorhexis after surgical correction: wide, regular, and not overlapping the optic of the IOL.

Postoperatively, the patient reported progressive improvement in visual quality. At 1 week postoperative, she described a marked reduction of ND and high overall satisfaction. At the 1-month follow-up, we report complete resolution of negative dysphotopsia and was satisfied with the procedure. A wide and well-centered capsulorhexis was achieved, with no overlap of the optic of the IOL (**Figure 3**). Best-corrected visual acuity remained stable, and no further surgical intervention was required. IOL explantation was definitely avoided.

## Discussion and conclusions

### Discussion

EDOF and multifocal IOL intolerance are multifactorial and may result from optical design, residual refractive error, ocular surface disease, neuroadaptation failure, or anatomical factors (7). Dysphotopsia remains after residual refractive error and ocular surface disease, an important factor in post-cataract surgery complaints (7–9). While explantation may be necessary in selected cases, identifying reversible causes is essential to minimize surgical risk and increase patient satisfaction.

ND cause remains unclear, associated with several predisposing factors, suggesting a multifactorial etiology. Regarding the material and optical design of the IOL, ND was initially associated with sharp-edged acrylic intraocular lenses (IOLs) with higher refractive indices (10–14). Ray-tracing analyses by Holladay identified a “type 3 shadow” caused by posterior refraction at sharp IOL edges, with higher refractive index acrylic lenses producing a wider anatomic range for shadow formation than silicone lenses. Rounded edges have been shown to disperse light and reduce shadow perception, though ND may still occur if the edge radius is small (15). Larger distance between the back of the iris and the anterior capsule of the lens can also predispose to ND (16). Indeed, Holladay calculated that the distance range that allows shadow to fall onto the retina is significantly larger for acrylic IOLs (0.06 to 1.23 mm) as compared with silicone IOLs (0.06 to 0.62 mm). Although several published cases support the role of material and design in ND, several publications of ND with low-index materials and round-edged IOLs also suggest that material and design are contributors rather than a definite cause (17, 18).

Another proposed factor for ND was reflection of the anterior capsulotomy edge onto the nasal retina, with symptom improvement observed when the IOL optic was positioned anterior to the capsulotomy, for example, with piggyback lens implantation (6, 17). Ray-tracing analyses supported an interaction between the anterior capsule and the IOL optic as a major contributing factor, rather than the refractive index of the IOL material or the depth of the posterior chamber (17).

Optical modeling indicates that several shadow mechanisms may occur. Holladay et al. (15) proposed three types of shadows that create negative dysphotopsia. One mechanism relates to internal light reflection within the IOL, which tends to produce peripheral visual disturbances associated with positive dysphotopsia. Another mechanism involves a discontinuity at the anterior edge of the optic, particularly when the lens sits deep in the posterior chamber. A third mechanism, more frequently implicated in negative dysphotopsia, results from light refraction at a sharp posterior edge of the optic, producing a penumbral shadow on the nasal retina. This effect is influenced by pupil size, the refractive index of the lens material, and the axial position of the IOL.

A small or irregular capsulorhexis may induce optic overlap and alter the functional performance of EDOF and multifocal IOLs. Adequately sized capsulorhexis provides uniform coverage of the optic edge, preventing stray light rays from entering the optical system and reaching the retina, thereby reducing total internal reflection and, therefore, positive and negative dysphotopsia (15), emphasizing the importance of appropriate capsulorhexis sizing and regularity. Optical modeling and clinical studies demonstrate that the size and configuration of the capsulorhexis, especially when small or irregular, can interact with the intraocular lens (IOL) to create an illumination gap or temporal shadow, characteristic of ND (19). Ray-tracing studies confirm that this interaction can result in a gap between rays refracted by the IOL, manifesting as ND (19).

Potential risks of anterior capsulorhexis revision include capsular extension with compromise of capsular bag stability, zonular stress or dehiscence, intraocular lens decentration, posterior capsule rupture, vitreous prolaps, and cystoid macular edema^XX^.

In this case, addressing the anatomical issue by removing the capsular fibrotic ring, enlarging the capsulorhexis, and regularizing the edge, aiming for a curvilinear form, resulted in symptom resolution, highlighting the importance of meticulous anterior segment evaluation in unsatisfied patients with multifocal IOLs. Early improvement of the reported symptoms suggests an immediate optical or geometric change in anterior capsule–optic interaction, while sustained resolution supports a stable anatomical effect. While capsulorhexis configuration has been implicated in ND, its direct surgical correction as a primary alternative to explantation remains underreported.

Strengths of this case include the detailed anterior segment assessment identifying a potentially reversible anatomical cause of persistent negative dysphotopsia and the successful application of a lens-preserving surgical approach. This case highlights a practical, reproducible intervention that directly addresses the anterior capsular configuration rather than proceeding with intraocular lens exchange.

Limitations include the single-case design, lack of objective quantification of dysphotopsia severity, and absence of standardized patient-reported outcome measures. Further studies are required to determine the reproducibility of this approach and to better define anatomical predictors of success.

In patients with persistent negative dysphotopsia and a well-centered IOL, careful evaluation of capsulorhexis configuration is essential, as surgical revision may resolve symptoms and avoid explantation.

### Conclusions

Capsulorhexis enhancement combined with removal of fibrotic areas in the anterior capsule can be a safe and effective, lens-preserving alternative to IOL explantation in selected patients with IOL intolerance due to ND. Early recognition of this anatomical factor may prevent unnecessary surgery. Careful anterior segment evaluation should be considered as part of the diagnostic workup of postoperative dysphotopsia to identify candidates for this targeted intervention.

## Data Availability

The original contributions presented in the study are included in the article/supplementary material. Further inquiries can be directed to the corresponding author.
